# Circadian Phase Preference in Pediatric Bipolar Disorder

**DOI:** 10.3390/jcm3010255

**Published:** 2014-03-11

**Authors:** Kerri L. Kim, Alexandra B. Weissman, Megan E. Puzia, Grace K. Cushman, Karen E. Seymour, Ezra Wegbreit, Mary A. Carskadon, Daniel P. Dickstein

**Affiliations:** 1 PediMIND Program at E.P. Bradley Hospital and the Alpert Medical School, Brown University, Providence, RI 02915, USA; E-Mails: aweissman@lifespan.org (A.B.W.); mpuzia@lifespan.org (M.E.P.); gcushman@lifespan.org (G.K.C.); kseymou2@jhmi.edu (K.E.S.); ezra_wegbreit@brown.edu (E.W.); daniel_dickstein@brown.edu (D.P.D.); 2 Chronobiology and Sleep Program at E.P. Bradley Hospital and the Alpert Medical School, Brown University, Providence, RI 02915, USA; E-Mail: mary_carskadon@brown.edu; 3 Centre for Sleep Research, University of South Australia, Adelaide 5005, Australia

**Keywords:** child, adolescent, bipolar disorder, circadian

## Abstract

Pediatric bipolar disorder (BD) rates have notably increased over the past three decades. Given the significant morbidity and mortality associated with BD, efforts are needed to identify factors useful in earlier detection to help address this serious public health concern. Sleep is particularly important to consider given the sequelae of disrupted sleep on normative functioning and that sleep is included in diagnostic criteria for both Major Depressive and Manic Episodes. Here, we examine one component of sleep—*i.e.*, circadian phase preference with the behavioral construct of morningness/eveningness (M/E). In comparing 30 BD and 45 typically developing control (TDC) participants, ages 7–17 years, on the Morningness-Eveningness Scale for Children (MESC), no between-group differences emerged. Similar results were found when comparing three groups (BD−ADHD; BD+ADHD; TDC). Consistent with data available on circadian phase preference in adults with BD, however, we found that BD adolescents, ages 13 years and older, endorsed significantly greater eveningness compared to their TDC peers. While the current findings are limited by reliance on subjective report and the high-rate of comorbid ADHD among the BD group, this finding that BD teens demonstrate an exaggerated shift towards eveningness than would be developmentally expected is important. Future studies should compare the circadian rhythms across the lifespan for individuals diagnosed with BD, as well as identify the point at which BD youth part ways with their healthy peers in terms of phase preference. In addition, given our BD sample was overall euthymic, it may be that M/E is more state *vs*. trait specific in latency age youth. Further work would benefit from assessing circadian functioning using a combination of rating forms and laboratory-based measures. Improved understanding of sleep in BD may identify behavioral targets for inclusion in prevention and intervention protocols.

## 1. Introduction

Although the incidence of mania was once considered negligible in childhood and adolescence, rates of pediatric bipolar disorder (BD) have notably increased over the past three decades. For example, the percentage of children and adolescents discharged from psychiatric hospitals in the U.S. with a diagnosis of BD surged from less than 10% in the mid-1990s to more than 20% in the mid-2000s [1]. The growing prevalence of pediatric BD is especially concerning given the significant morbidity and mortality associated with BD, including high rates of suicidality, academic and social impairment, lower perceived quality of life, and high health care expenditures [2,3,4].

Despite increasing data supporting pharmacological and psychotherapy treatment options for youth once diagnosed with BD, efforts are needed to identify factors useful in earlier detection to help address this serious public health concern. Towards that end, sleep is of particular interest given the sequelae of disrupted sleep on daily psychosocial functioning (e.g., poor academic performance, decreased emotion/behavior regulation [5,6]) and that sleep alterations are included in the Diagnostic and Statistical Manual, 5th edition [7] criteria for both Major Depressive and Manic Episodes. Data exist not only documenting sleep disturbances (e.g., insomnia, irregular sleep schedules) as common among BD youth, but also indicating its importance as a prodrome [8,9]. In a recent study of 82 youth diagnosed with BD, for example, nearly half of parents retrospectively reported sleep disruption (e.g., insomnia, parasomnias) as the first observed symptom [10]. Furthermore, in comparing 100 youth at high-risk for BD by virtue of having a parent diagnosed with BD Type I to 112 healthy controls with no parental history of BD, Shaw and colleagues [11] showed that decreased need for sleep differentiated those at high- *vs*. low-risk during a 10-year follow-up period. Studies with adults diagnosed with BD also report that sleep disturbance (*i.e.*, induction of sleep deprivation) may contribute to relapse/the onset of subsequent mood episodes [12]. In fact, interpersonal and social rhythm therapy (IPSRT) [13] was developed based upon the adult literature showing erratic sleep-wake cycles in BD and with the goal of increasing consistency in an individual’s sleep and daily functioning as a means to minimize risk of symptom recurrence.

Borbély’s [14] widely recognized two-process model of sleep provides a framework to advance what is known about sleep in childhood. Within this framework, the homeostatic component (Process S) regulates the need for sleep and the circadian component (Process C) manages the timing of sleep onset and offset. Interestingly though, studies-to-date focused on the outcomes of disrupted sleep in children are best tied to the model’s homeostatic component (Process S) as researchers specifically investigate the role of sleep loss, decreased sleep quality, and subsequent daytime sleepiness. Less is known about the model’s circadian component (Process C) in children’s psychosocial outcomes.

The endogenous circadian system can be studied with intensive and sometimes invasive lab-based measures (e.g., core body temperature, melatonin and cortisol levels, forced desynchrony) [15,16,17]. However, a more appropriate first step and less intensive proxy is to measure the behavioral construct of morningness/eveningness (M/E), which gauges an individual’s circadian phase preference by acknowledging the timing of optimal daily functioning [18,19]. While M/E is presumed to be normally distributed in the general population, studies suggest that adults with BD endorse more extreme evening phase preference (*i.e.*, time preference for sleep and wake—“lark” *vs*. “owl”) compared to their control counterparts (see [20] for a more detailed review of adult-specific findings) [21,22]. Further, Ashman *et al*. reported evidence of such phase delay in a sample of rapid cycling BD adults such that morning activities in BD adults were more delayed compared to controls [23]. Greater eveningness in BD adults is also associated with heightened clinical severity (e.g., lower global assessment of functioning scores and more self-reported symptoms of depression) [21,24]. Moreover, adults diagnosed with BD assigned to IPSRT for stabilization of mood symptoms went significantly longer during the two-year study without experiencing a new mood episode compared to those BD I adults assigned to a clinical management protocol (*i.e.*, provided psychoeducation and non-specific support) [25].

Despite existing studies documenting sleep dysfunction in children and adolescents with BD (e.g., decreased sleep duration, initial insomnia; see [8]), little is known about circadian phase preference specific to pediatric BD. To address this gap in knowledge, we evaluated the phase preference of youth with BD *vs*. typically developing control (TDC) participants without psychopathology. Given the dearth of such comparative studies, our study was intended as a first step towards understanding potential alterations in chronobiology associated with BD in children that might guide subsequent studies with lab-based measures. Based on the adult BD literature [18,19], we hypothesized that BD youth would have greater preferences for eveningness compared to TDC participants.

## 2. Methods

### 2.1. Participants

Participants ages 7 to 17 years were enrolled in an institutional review board-approved study performed at Bradley Hospital and Brown University. After the study was explained and before participation, parents and children gave written informed consent and assent. Participants were recruited through advertisements distributed to local physicians’ offices and placed on local/national websites.

BD (*N* = 30) inclusion criteria were: (1) Meeting DSM-IV-TR criteria for BD, including history of at least one episode of hypomania (>4 days) or mania (>7 days) wherein the child exhibited abnormally elevated or expansive mood as well as three or more DSM-IV criterion “B” mania symptoms; and (2) The presence of a primary caretaker to grant consent and participate in the research process. Children with irritability only, without elevated or expansive mood, were excluded from this group as were children with BD “not otherwise specified”. Thus, all BD participants met the criteria of Leibenluft *et al*. narrow-phenotype BD [26].

Additional exclusion criteria were: (1) A pervasive developmental disorder diagnosis; (2) Psychosis interfering with the child’s capacity to comply with study procedures; and IQ < 70 (the latter applied for both BD and TDC groups).

TDC (*N* = 45) inclusion criterion was a negative psychiatric history in the control participant and their first-degree relatives.

### 2.2. Procedure

Following a telephone interview to ascertain relevant symptoms, potential participants were invited for a more comprehensive on-site screening that included the Schedule for Affective Disorders and Schizophrenia for School-Aged Children, Lifetime Version (K-SADS-PL) administered by doctoral-level clinicians (M.D. or Ph.D.) with established inter-rater reliability (kappa ≥ 0.85; DPD, KLK) [27].

Current mood symptoms were assessed for the BD participants with the Young Mania Rating Scale (YMRS) and Children’s Depression Rating Scale—Revised (CDRS-R) [28,29].

The Wechsler Abbreviated Scale of Intelligence (WASI) was administered to every participant as an estimate of cognitive ability [30]. Youth completed several rating forms including the Conners’ Rating Scales—Revised (CRS-R) [31] and a self-report of pubertal development [32]. Parents also completed ratings of their child’s psychosocial functioning and provided demographic information. Socioeconomic status (SES) was categorized according to the Hollingshead Index [33].

### 2.3. Circadian Phase Preference

The Morningness-Eveningness Scale for Children (MESC) was the primary outcome measure for this study [34]. The MESC is a self-report measure that assesses the construct of morningness/eveningness (M/E) as a proxy for circadian phase preference and was based upon the accepted adult scale of M/E developed by Smith and colleagues [35]. The scale consists of 10 multiple-choice questions (e.g., “Your parents have decided to let you set your own bed time. What time would you pick?”) and asks youth to base their answers on how they have felt during the recent weeks. The MESC has been shown to have both adequate reliability and validity with children and adolescents ages 8 to 16 years [34,36,37]. Scores can range from 10 (extreme evening or E-type) to 43 (extreme morning or M-type). Internal reliability of the MESC (Cronbach’s alpha) for the current sample was 0.79 and scores ranged from 17 to 40. To better describe youth categorized as extreme chronotypes (*i.e.*, M- or E-type), cut-off scores were calculated based upon the 10th and 90th percentiles of the current TDC sample distribution in line with procedures established by previous research [38,39]. The 10th percentile of MESC scores = 22 and 90th percentile of MESC scores = 37.4.

### 2.4. Data Analytic Plan

We performed an analysis of covariance (ANCOVA) using group (BD, TDC) as the fixed factor and MESC scores as the dependent measure. Age was selected as the covariate based on its negative association with MESC scores in this sample (*r* = −0.34 to −0.52, *p* = 0.004 to 0.02), as well as the known role of age on circadian phase preference [40]. Pubertal development, also negatively associated with MESC scores in this sample (*r* = −0.32 to −0.38, *p* < 0.05), was not included as a covariate given its significant positive association with age so as to limit issues of multi-collinearity.

Given the wide range of ages represented in our sample, we performed exploratory ANOVAs using group as the fixed factor and MESC scores as the dependent measure with samples limited to latency age youth (<13 years old) and then adolescents (ages 13 years and older). Also, given the high rate of co-occurrence of BD and ADHD, we sub-divided the BD group into those with (BD+ADHD) and without (BD−ADHD) ADHD, and performed a post-hoc analysis of circadian phase preference via an ANCOVA with group as the fixed factor, age as the covariate, and MESC scores as the dependent variable.

## 3. Results

### 3.1. Participants

The sample included 75 youth ranging in age from 7 to 17 years: *N* = 30 in the BD group and *N* = 45 in the TDC group. There were no between-group differences in age, sex, or race/ethnicity (see Table 1). Regardless of diagnostic group, MESC scores were negatively correlated with pubertal status (*r* = −0.33, *p* < 0.01) and age (*r* = −0.41, *p* < 0.01), indicating that as youth developed/aged, their preferences shifted towards greater eveningness.

All youth in the BD group met criteria for type I BD (although type II was not exclusionary for this study). The BD group was overall euthymic by mood rating during study participation (CDRS_mean_ = 30.0 ± 11.7, YMRS_mean_ = 8.3 ± 7.3). Of the 30 BD youths, 23 (77%) were euthymic (YMRS ≤ 12, CDRS < 40), 2 (7%) were depressed (YMRS < 12, CDRS ≥ 40), 3 (10%) were hypomanic (YMRS = 13–24, CDRS < 40), and 2 (7%) were in a mixed mood state (YMRS > 12, CDRS ≥ 40) during study participation. Mood state for the BD group did not significantly correlate with MESC scores (*r* = −0.07, *p* = 0.73). CDRS and YMRS scores were also not independently associated with MESC scores among BD youths (*r*_CDRS_ = −0.01, *p* = 0.97 and *r*_YMRS_ = 0.22, *p* = 0.26).

**Table 1:** Demographic information for the Bipolar Disorder (BD) and typically developing control (TDC) groups.

Characteristic	BD Group (*N* = 30)	TDC Group (*N* = 45)	*F-*Value or *χ*^2^	*p*-Value
*Mean Age in Years (SD)*	13.55 (2.67)	12.68 (3.39)	1.38	0.24
*Gender, n (%)*			3.21	0.07
Males	19 (63)	19 (42)		
Females	11 (37)	26 (58)		
*Race, n (%)*			3.82	0.43
Caucasian	25 (83.3)	35 (77.8)		
African American	1 (3.3)	1 (2.2)		
Asian	0 (0)	2 (4.4)		
American Indian	0 (0)	0 (0)		
Multiracial	4 (13.3)	4 (8.9)		
Unknown	0 (0)	3 (6.7)		
*Ethnicity, n (%)*			3.00	0.22
Hispanic	0 (0)	4 (8.9)		
Non-Hispanic	29 (96.7)	38 (84.4)		
Unknown	1 (3.3)	2 (4.4)		
*Hollingshead Score, n (%)*			7.01	0.14
8–19	3 (10.0)	1 (2.2)		
20–29	3 (10.0)	3 (6.7)		
30–39	3 (10.0)	4 (8.9)		
40–54	14 (46.7)	14 (31.1)		
55–66	6 (20.0)	21 (46.7)		
Not reported	1 (3.3)	2 (4.4)		
*Secondary Diagnoses, n (%)*				
ADHD	20 (66.7)			
Oppositional Defiant	21 (70.0)			
Obsessive-Compulsive	2 (6.7)			
Generalized Anxiety	3 (10.0)			
Specific Phobia	6 (20.0)			
Separation Anxiety	2 (6.7)			
Social Anxiety	2 (6.7)			
Agorophobia	2 (6.7)			
Panic	1 (3.3)			
Tic	5 (17.2)			
*Medication Free, n (%)*	4 (13.8)			
*Medication Type, n (%)*				
Lithium	10 (33.3)			
Atypical Antipsychotic	19 (63.3)			
Anti-Epileptic	6 (20.0)			
Antidepressants	6 (20.0)			
Stimulants	11 (36.7)			
Non-Stimulant/ADHD	6 (20.0)			

### 3.2. Primary Analysis

ANCOVA assessing between-group differences in circadian phase preference while controlling for age did not show a main effect of diagnostic group on the MESC [*F*(1,71) = 1.49, *p* = 0.23, partial eta^2^ = 0.02]. Based on 10th (MESC scores < 22; E-types) and 90th (MESC scores > 37.4; M-types) percentiles calculated for our TDC sample, the BD (*M*_mesc_ = 27.35 *±* 4.77) youth were best characterized as intermediate types, such that they did not endorse strong tendencies toward morningness or eveningness. In fact, only 3 BD participants described themselves as E-types, and only 1 BD participant described themselves as M-types.

### 3.3. Post-Hoc Analyses

Post-hoc analyses to further evaluate the effect of age showed a significant between-group difference on MESC [*F*(1,39) = 4.70, *p* < 0 .04, partial eta^2^ = 0.11] when the sample was limited to adolescents (13 years or older). Specifically, BD teens (*n* = 17) reported significantly greater eveningness (*M*_mesc_ = 25.00 *±* 4.26) compared to TDC teens (*n* = 24; *M*_mesc_ = 28.13 *±* 4.74). There was no between-group difference on MESC [*F*(1,25) = 0.36, *p* *=* 0.55, partial eta^2^ = 0.01] when the sample was limited to latency age (<13 years old) BD (*n* = 6; *M*_mesc_ = 32.17 *±* 3.54) or TDC (*n* = 21; *M*_mesc_ = 30.76 *±* 5.34) youth.

Post-hoc analyses to evaluate the effect of comorbid ADHD among age- and gender-matched groups of BD+ADHD, BD−ADHD, and TDC participants (*N* = 10 per group) did not show significant between-group differences on the MESC [*F*(2,26) = 0.58, *p* = 0.57, partial eta^2^ = 0.04], nor did MESC scores significantly correlate with CRS-R ADHD Index scores (*r*_BD+ADHD_ = 0.06, *p* = 0.88 and *r*_BD−ADHD_ = −0.12, *p* = 0.79).

## 4. Discussion

Our study is an initial attempt to understand the circadian rhythms of youth with BD. Contrary to our hypothesis that BD youth would be characterized by greater eveningness compared to TDC participants, we did not find significant between-group differences in self-reported MESC scores when comparing the entire sample of BD youths to the entire sample of TDC participants. Restricting our sample to just teenagers, but not latency-age youth, however, showed that BD participants had significantly greater eveningness than TDC participants. Additional post-hoc analyses did not demonstrate a significant effect of ADHD comorbidity when sub-dividing the BD sample into participants with and without comorbid ADHD. Caution is urged in over-interpreting these latter results, given potential type I and II errors, as well as the need to corroborate self-reported information with other biological assessments of circadian rhythms, such as dim light melatonin onset phase.

While perhaps not a trait marker specific to all of pediatric BD given the null results when including a wide age range of participants, it is possible that M/E transitions into a more important role as youth with BD age. In line with extensive efforts documenting the general phase delay common to adolescence, we found a negative association between MESC scores and age, as well as pubertal status for the overall sample [18,39,41]. We then found that BD teens, but not latency-age youth, are consistent with circadian phase data available for adults with BD as they reported significantly greater eveningness compared to TDC teens. For example, Ahn *et al*. and Wood *et al*. reported lower M/E scores among BD adults compared to TDC adults, reflecting circadian phase delay and greater eveningness preferences [21,22]. The current finding suggests an exaggerated response of a developmentally expected change—much like the assumption that psychopathology represents an exaggeration of normal traits [42]. Future studies are needed to compare circadian functioning across the lifespan for individuals diagnosed with BD to better understand the timing of this exaggerated transition towards greater eveningness, as well as the possible implications of intervening on an adolescent’s phase delay on their adult course of BD.

In addition to identifying the timing at which the shift towards greater eveningness for BD youth exceeds that of their TDC peers, further exploration of M/E as a trait *vs*. state phenomenon is likely important for future studies. Wood and colleagues found that M/E scores were negatively associated with depression ratings and positively associated with global assessment of functioning scores for their BD group, suggesting that greater eveningness is linked to current depression severity and impairment in adults. Similarly, Ashman and colleagues found that, while morning activities were delayed in BD adults compared to controls, the activities were notably more delayed during periods of depression compared to (hypo)mania [23]. It is possible that our finding that current depression symptoms of BD youth did not correlate with MESC scores is related to our inclusion of those who were generally euthymic during study participation. Perhaps then greater circadian preference towards extreme E-type reported during euthymic states is a marker for the severity of sleep disruption possible during mood episodes. Additional studies using longitudinal designs and including BD youths across mood states (mania, depression, and euthymia), similar to the work of Leibenluft’s group who followed a sample of BD adults over 18-months to naturalistically assess the interaction of mood and sleep variables (including duration), are warranted to determine the timing or onset of sleep problems in children and adolescents with BD, given that such sleep disturbances are among the most common prodrome of mania among BD adults [8,12,43].

Our study has several limitations. First, our study relied on subjective report forms of circadian phase preference and psychiatric symptoms. Given that this is the first step in understanding sleep and circadian rhythms in BD youths, future work should pair questionnaires with laboratory-based or more objective methods of assessment. Actigraphy specifically has proven a useful tool in capturing sleep and circadian rhythm data with BD adults within their naturalistic settings. For example, Jones and colleagues found BD adults to have greater variability in their circadian patterns compared to control participants [44]. Gathering such data from BD teens might afford better understanding of how their general circadian phase preference interacts with actual sleep behavior (e.g., sleep onset consistency on school *vs*. weekend days) to impact mood and psychosocial functioning [45]. Future studies should also include evaluation of genetic moderators, given the link between psychiatric symptoms, circadian phase preference (M- *vs*. E-type), and genes previously implicated in circadian clock function. For example, the CLOCK gene may be of particular interest, as past findings have shown that BD adults with the C allele of CLOCK3111 T/C demonstrate greater eveningness, delayed sleep onset, and decreased total sleep [46].

The high rate of co-morbid ADHD in our BD group is another limitation. However, our sample’s level of ADHD comorbidity aligns with prior studies, and thus findings may generalize better than if we had excluded BD youths with comorbid ADHD [9,47]. To address this potential limitation, we conducted post-hoc analyses of BD youths with and without comorbid ADHD. These analyses failed to reveal M/E differences among youth with BD without comorbid ADHD, BD with comorbid ADHD, and TDC, possibly due to type II error and reflective of the small group sizes (*N* = 10 in each). Future studies are needed to explore the circadian timing system among larger diagnostic groups. Moreover, available data on circadian functioning indicate delayed phase preference in ADHD children and adults who report difficulties with sleep onset and offset, with greater eveningness positively associated with symptom severity, particularly increased inattention [48]. This association was not found in the current sample, as MESC scores did not significantly correlate with CRS-S scores. Future studies might clarify this association further by including teacher report of behavior and direct observation, as well as by evaluating the independent and joint impact of endorsed M/E and actual sleep behavior. The latter is highlighted because data suggest the most common subjectively-reported sleep issues in ADHD are irregular sleep patterns, delayed sleep onset, and longer nighttime duration of sleep [49,50,51].

## 5. Conclusions

In summary, contrary to our hypothesis, we did not find between-group differences in circadian phase preference among BD and TDC youths when including a wide pediatric age range. However, BD adolescents endorsed significantly greater eveningness compared to their TDC peers—a finding consistent with BD adult-based circadian phase data. Future work is needed to identify the point at which BD youth part ways with their non-psychiatric peers and begin to demonstrate this exaggerated shift towards eveningness, as well as the possible ways in which an individual’s course of BD is altered if their phase delay is addressed sooner rather than later. Also, to determine if M/E is a state *vs*. trait phenomenon, future work is needed to determine the role of mood state in circadian phase preference by studying BD youths while currently euthymic, manic, and depressed and to longitudinally assess the role of sleep in the prediction of mood episodes and their severity.
